# Delayed Spleen Rupture After Trauma

**DOI:** 10.7759/cureus.106433

**Published:** 2026-04-04

**Authors:** Mohamed Ahmed, Kim Nguyen, Stylianos Tsintzilonis, Mohammed Asaad, Danya Auda

**Affiliations:** 1 Surgery, University of California, Riverside, Riverside, USA; 2 Surgery, AdventHealth Tampa, Tampa, USA; 3 General Surgery, AdventHealth Tampa, Tampa, USA; 4 Psychology, University of California, Riverside, Riverside, USA

**Keywords:** anemia, left sided abdominal pain, rib fractures, spleen hemorrhage, splenic trauma

## Abstract

The spleen is the most commonly injured solid organ in patients sustaining blunt abdominal trauma. The widespread use of computed tomography (CT) has significantly improved its diagnosis and grading. Delayed splenic rupture (DSR) refers to hemorrhage occurring more than 48 hours after blunt abdominal trauma in a patient who was initially hemodynamically stable. Although uncommon, it remains a recognized complication of splenic injury and can occur days to weeks after the initial trauma and carries significant mortality. We present a case of DSR presented one week after the injury, which was managed nonoperatively. The aim is to shed light on this uncommon but potentially fatal complication of spleen injury.

## Introduction

The majority of splenic ruptures occur acutely following blunt abdominal trauma; however, a minority of patients present with delayed splenic rupture (DSR), which may manifest days to weeks after the initial injury [1]. DSR, first described by Baudet in 1907 [2], is defined as splenic hemorrhage occurring more than 48 hours after trauma in a previously hemodynamically stable patient [3,4]. 

Although relatively uncommon, DSR is associated with a higher mortality rate (5-15%) compared with the approximately 1% mortality rate observed in acute splenic injuries [5]. Multidetector computed tomography (MDCT) is the preferred imaging modality for detecting splenic injuries, with sensitivity and specificity approaching 95% [6]. Despite advances in imaging and nonoperative management of splenic injuries, DSR remains a potentially life-threatening complication that requires a high index of clinical suspicion. Here, we present a case of DSR following blunt abdominal trauma and review the relevant literature.

## Case presentation

**Figure 1:**
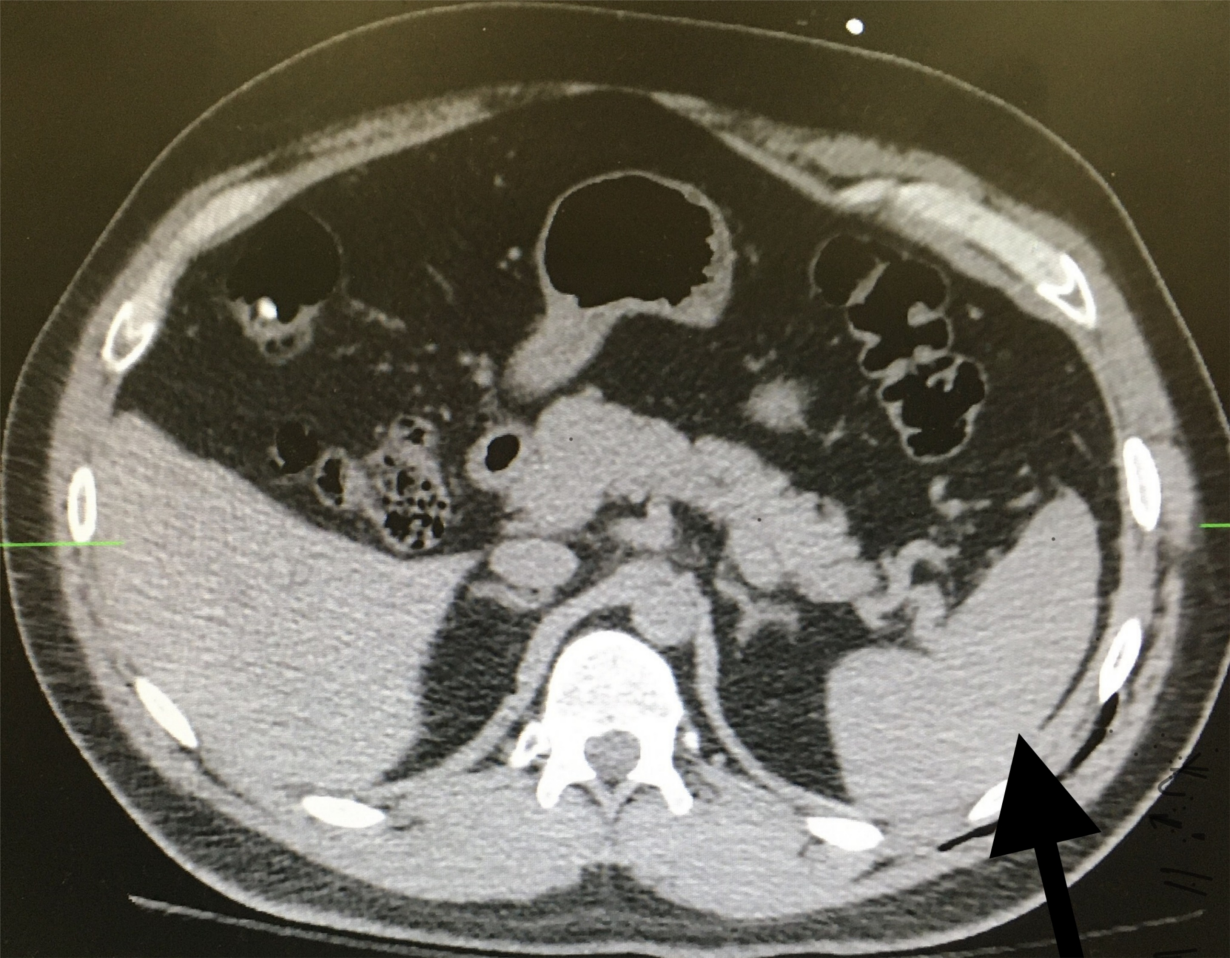
Computerized tomography of the abdomen at the first presentation No evidence of spleen injury (black arrow).

A 36-year-old male patient presented to the emergency department with left lower chest pain after tripping while standing on the bed of his truck and sustaining a fall. On arrival, he was hemodynamically stable, and initial laboratory studies were within normal reference ranges. Computed tomography (CT) scan of the chest, abdomen, and pelvis demonstrated an acute fracture of the left sixth rib with an associated pulmonary contusion. No evidence of splenic injury or intra-abdominal bleeding was identified (Figure 1). During this initial admission, the patient was managed with analgesia and pulmonary supportive care and was discharged after 48 hours in stable condition.

**Figure 2:**
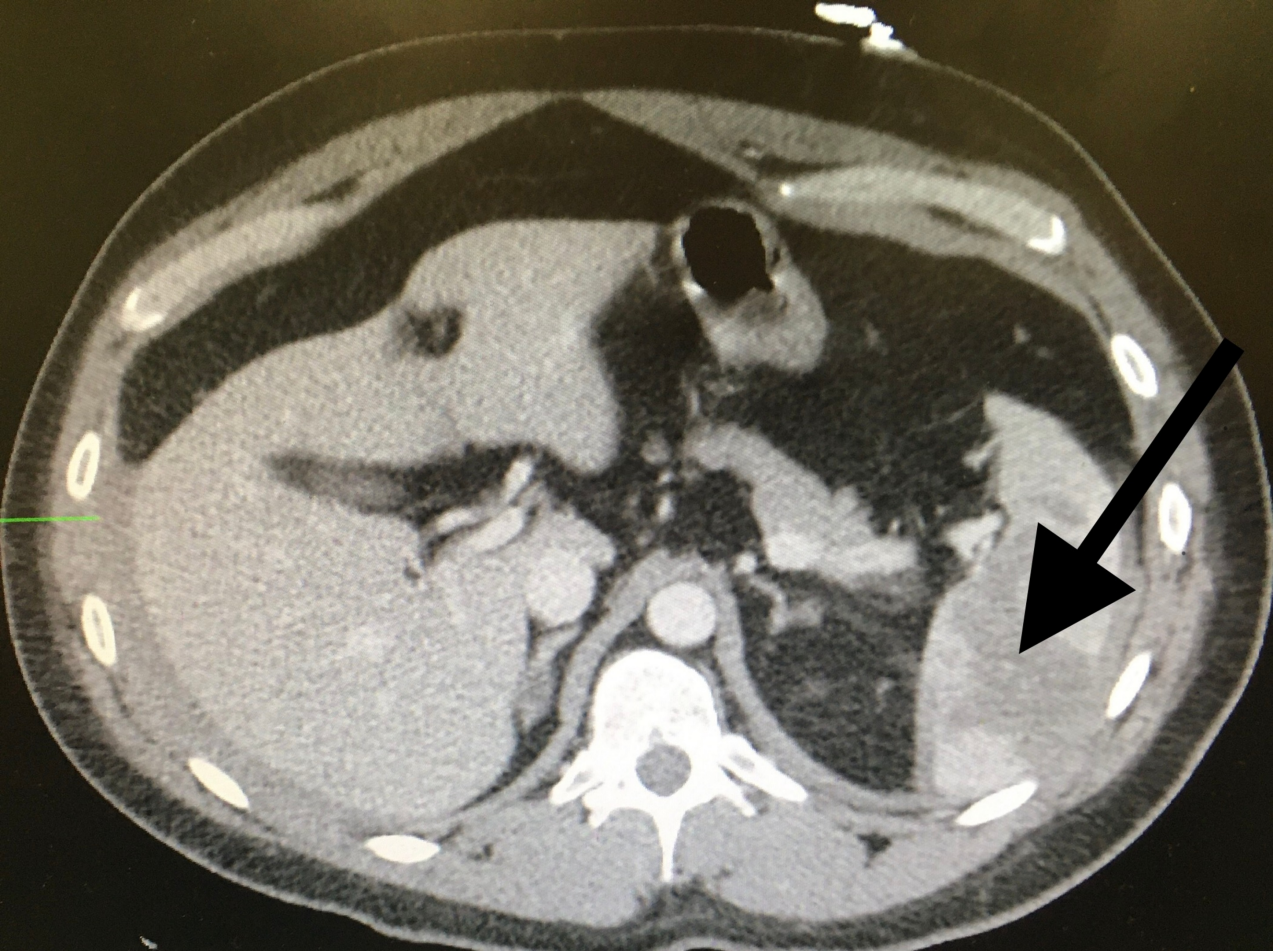
Computerized tomography of the abdomen one week later Grade 3 spleen injury (black arrow).

Seven days later, he returned to the emergency department with complaints of left upper quadrant abdominal pain and dizziness. Laboratory evaluation revealed a hemoglobin level of 10.3 g/dL, decreased from 16.0 g/dL during the prior admission (reference range 13.5-18.0 g/dL). A contrast-enhanced CT scan of the abdomen and pelvis revealed a deep parenchymal laceration (>3 cm) consistent with grade 3 splenic injury (Figure 2).

**Figure 3:**
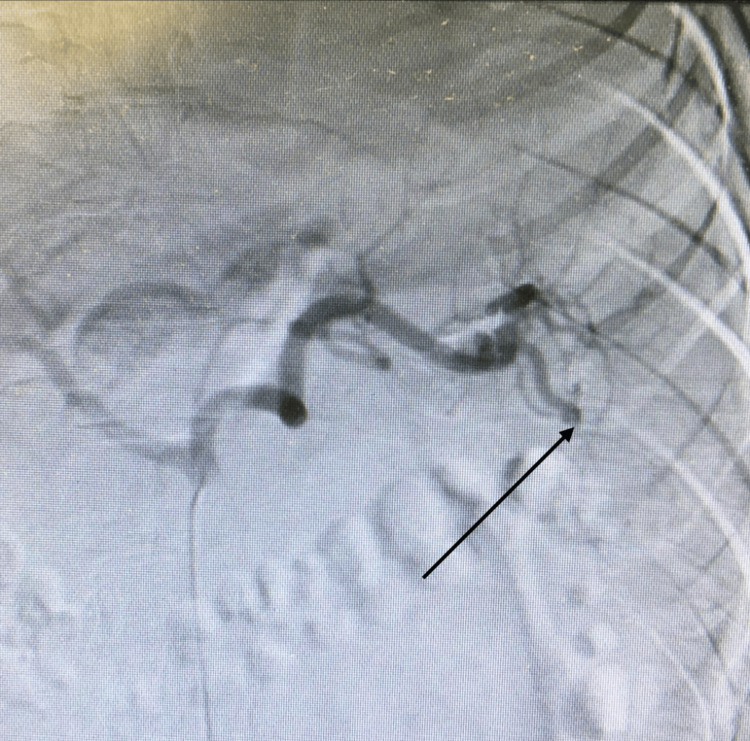
Arteriography of the splenic artery Lower pole branch of the splenic artery (black arrow)

In the absence of hemodynamic instability, the patient was managed nonoperatively with close observation, serial abdominal examinations, and hemoglobin monitoring per institutional protocol. Interventional radiology was consulted. Celiac and splenic angiography showed no active contrast extravasation or pseudoaneurysm. Given the grade III injury and significant hemoglobin drop, selective distal splenic artery embolization was performed the day of the second admission: the middle and lower segmental branches were catheterized and embolized with geofoam to near-stasis, with preservation of proximal flow to the superior pole (Figure 3).

## Discussion

In blunt abdominal trauma, the spleen is the most commonly injured solid organ, and such injuries carry substantial morbidity and mortality [7]. Beyond acute, life-threatening hemorrhage, DSR, pseudocysts, and abscesses may complicate the course of splenic injury [8]. DSR is classically defined as a clinically significant splenic hemorrhage occurring >48 hours after trauma. It is reported in 1-5% of splenic injuries, most often between days 4-8, and carries a higher mortality (~5-15%) than acute injury (~1%) [9]. 

Over the past five decades, management has shifted from routine laparotomy to selective nonoperative management in hemodynamically stable patients, a change enabled by modern CT imaging for diagnosis and grading. The classic radiographic triad associated with blunt splenic rupture (elevation of the left hemidiaphragm, left lower lobe atelectasis, and left pleural effusion) is often absent and cannot be considered a reliable diagnostic indicator. However, elevation of the left hemidiaphragm following blunt trauma should always raise suspicion for a possible occult splenic injury until proven otherwise [10]. 

The pathophysiology of DSR has been explained by several proposed theories, including increased capsular pressure secondary to clot lysis and a rise in oncotic pressure leading to rupture of the splenic capsule; tamponade of a peri splenic hematoma by surrounding organs or the momentum with subsequent rupture into the peritoneal cavity once containment is lost; and delayed bleeding due to rupture of a post-traumatic intraparenchymal pseudoaneurysm or splenic pseudocyst [11]. In our case, it is presumed to be an evolution of an initial contusion or micro-laceration. While routine repeat imaging after blunt splenic injury is not universally recommended, several guidelines advise targeted reimaging only in patients who: (i) develop or worsen anemia, (ii) have high-grade splenic injury and/or a large subcapsular hematoma, (iii) have underlying splenic pathology or coagulopathy, or (iv) cannot be reliably followed clinically [12]. As nonoperative management has become the standard approach for hemodynamically stable patients, new patterns and timing of post-injury complications have become increasingly recognized [13].

DSR has been reported in approximately 5% of adults managed nonoperatively after splenic trauma. Other delayed complications include post-traumatic splenic pseudocyst formation (approximately 0.44% of cases) and, less commonly, splenic abscess formation following blunt trauma [14]. Management of DSR is guided by the patient’s hemodynamic status and imaging findings. Hemodynamically unstable patients typically require urgent operative intervention, most commonly splenectomy. In contrast, stable patients may be managed nonoperatively with close observation and selective splenic artery angioembolization when indicated. Routine radiographic follow-up is not universally required in stable patients but may be considered in those with higher-grade injuries, after angioembolization, or when new or worsening symptoms develop [15].

Maintaining a high index of suspicion is essential for early detection of delayed splenic rupture in patients who sustained a high-impact mechanism of injury, including high-speed motor vehicle collision, fall from 10 feet height, crush injury, auto-pedestrian collision, trauma consistent with significant energy transfer, and increased risk for severe internal injury. Patients who sustain high-impact injuries or have concomitant injuries to adjacent organs may benefit from follow-up MDCT within two to three days after the initial trauma or before hospital discharge to identify evolving splenic injury or delayed complications [16].

## Conclusions

Delayed complications of blunt splenic trauma may present outside trauma centers and should remain a diagnostic consideration in patients with recent abdominal injury. A high index of suspicion is essential for early recognition of DSR. The shift toward nonoperative management in hemodynamically stable patients has been facilitated by the widespread use of contrast-enhanced CT, which accurately characterizes injury patterns, grades splenic injury severity, and identifies clinically significant findings that guide management decisions. Patients who sustain high-impact injuries or have associated injury to adjacent organs may benefit from follow-up MDCT within two to three days after the initial trauma or before hospital discharge to detect evolving splenic injury or delayed complications.
